# Continuous training and certification in neonatal resuscitation in remote areas using a multi-platform information and communication technology intervention, compared to standard training: A randomized cluster trial study protocol

**DOI:** 10.12688/f1000research.12269.3

**Published:** 2018-03-08

**Authors:** Carlos Alberto Delgado, Enrique M. Gómez Pomar, Pablo Velásquez, Víctor Sánchez, Roberto Shimabuku, Luis Huicho, RCPNEOPERU Study Group

**Affiliations:** 1 Department of Pediatrics, School of Medicine, Universidad Nacional Mayor de San Marcos, Lima, Peru; 2 Neonatal Unit, Instituto Nacional de Salud del Niño, Lima, Peru; 3 Centro de Investigación para el Desarrollo Integral y Sostenible (CIDIS) and Centro de Investigación en Salud Materna e Infantil (MAMAWAWA), Universidad Peruana Cayetano Heredia, Lima, Peru; 4 Department of Pediatrics, Division of Neonatology, University of Kentucky, Lexington, KY, 40506, USA; 5 Neonatal Unit, Instituto Nacional Materno Perinatal, Lima, Peru; 6 School of Medicine, Universidad Peruana Cayetano Heredia, Lima, Peru

**Keywords:** In service training, Neonatal resuscitation, Cluster randomized trial

## Abstract

**Background**: About 10% of all newborns may have difficulty breathing and require support by trained personnel. In Peru, 90% of deliveries occur in health facilities. However, there is not a national neonatal resuscitation and certification program for the public health sector. In addition, the Andes and the Amazon regions concentrate large rural remote areas, which further limit the implementation of training programs and the accomplishment of continuous certification. Neonatal resuscitation training through the use of information, communication and technology (ICT) tools running on computers, tablets or mobile phones, may overcome such limitations. This strategy allows online and offline access to educational resources, paving the way to more frequent and efficient training and certification processes.

**Objective**: To evaluate the effects of a neonatal resuscitation training and certification program that uses a multi-platform ICT (MP-ICT) strategy on neonatal health care in remote areas.

**Methods**: We propose to conduct the study through a cluster-randomized trial, where the study and analysis unit is the health care facility. Eligible facilities will include primary and secondary health care level facilities that are located in provinces with neonatal mortality rates higher than 15 per 1,000 live births. We will compare the proportion of newborns with a heart rate ≥100 beats per minute at two minutes after birth in health care facilities that receive MP-ICT training and certification implementation, with those that receive standard training and certification.

**Discussion**: We expect that the intervention will be shown as more effective than the current standard of care. We are prepared to include it within a national neonatal resuscitation training and certification program to be implemented at national scale together with policymakers and other key stakeholders.

**Trial registration**: ClinicalTrials.gov Nº NCT03210194

**Status of the study**: This study is enrolling participants by invitation only.

Study protocol version 1.1 – March 31st, 2017

## Introduction

### Background

Approximately 10% of newborns may have difficulty breathing at birth and require urgent support from trained personnel ^1^. The first 60 seconds of life in those conditions require immediate and effective interventions to help infants survive ^2^. Unfortunately, some newborns in low- and middle-income countries do not have access to appropriately trained personnel, making asphyxia one of the leading causes of neonatal death in those settings ^3^.

In Peru, about 550,000 births occur per year (Peruvian Institute for Statistics and Informatics 2015; https://www.inei.gob.pe), and this means that approximately 6 newborns each hour may require respiratory support at birth. Problems in training health workers for adequate support of newborns is aggravated by a high turnover rate of personnel, particularly in small cities and rural areas, which hampers regular conduction of local training programs, even if they are perceived as a positive incentive ^4^. Peru is a high-middle-income country that has made significant progress in reducing neonatal and infant mortality in the last decade, despite inequities in access to health services, especially in rural areas of the Amazon and the Andes ^5, 6^. In the years 2011–2012, the Peruvian neonatal mortality rate was estimated at 12.8 per thousand live births ^7^, with an estimated 20 per thousand live births for Pasco and Cusco regions, 15 for Ayacucho and Amazonas, 12 for Huancavelica and 8 for Lima ^8^. From 2000 to 2013, neonatal mortality in Peru fell by 51% from 16.2 deaths per 1000 livebirths to 8.0 ^9^.

The Neonatal Resuscitation Program (NRP) from the American Academy of Pediatrics, includes evidence-based clinical guidelines and was designed to train health care providers and instructors in hospital settings. The NRP is a paid course that has 2 components, an online component (internet-dependent) and a practice/evaluation component ^1, 10^. A variant of the NRP program is the Helping Babies Breath (HBB) program, which was designed to provide neonatal resuscitation training in resource-poor settings. The HBB has shown to reduce neonatal mortality and it is proposed as a successful global intervention ^11^. However, the greatest limitation of these programs is the need for recurrent training with a frequency of less than 12 months to maintain the acquired skills and knowledge ^12^.

One of the few neonatal resuscitation programs that has been successfully implemented outside the United States is the Neonatal Resuscitation Program from the Brazilian Society of Pediatrics, which was implemented in 1991 (Brazilian Society of Pediatrics. Neonatal Resuscitation Program (Brazilian NRP); http://www.sbp.com.br/reanimacao). In Peru, despite several attempts made by the Social Security (Essalud) and the Ministry of Health ^13– 16^, there is still no national neonatal resuscitation program in place.

A training system using multi-platform information, communication and technology (MP-ICT) offers reliable and easier access to information and training packages from different locations, which can be implemented even in rural settings without access to the internet ^17^. Platforms may be available online, and users can download the training content through a PC, tablet or mobile phone ^18^. The training content may be repeatedly available after download, even in remote areas without access to internet.

Our study aims to evaluate the performance of a continuous process of training and certification in neonatal resuscitation in primary and secondary health facilities in the departments of Ayacucho and Cusco, through the use of MP-ICT. It aims at providing proof of concept of the intervention, which then could be incorporated as a component of a national neonatal resuscitation program to be implemented nationwide.

### Choice of comparator

The traditional way to train and certify health care professionals is through administration of a conventional theoretical course, reinforced subsequently by a practical component. This modality requires face-to-face contact between trainers and trainees. Health workers need to move from their job site to the instruction sites, usually located in the capital city of the department or in the capital city of the country. This poses economic and logistic challenges to both health authorities and health workers, and results in erratic frequency and low uptake of training courses.

Since most of the deliveries in our study region are attended by nurses and midwives, and there is a scarcity of local based doctors, we are going to use hospital based instructors as NRP trainers. This traditional training was considered as standard control, and it has been selected as the comparator for our MP-ICT intervention, because a comparison using placebo or no-training was considered to be unethical.

### Research hypothesis

The use of MP-ICT for training and certification in neonatal resuscitation will increase the proportion of children with heart rate equal or greater than 100 beats per minute at two minutes of life, as compared to standard training.

### Primary objective

To evaluate the effects of the use of a MP-ICT for training and certification on neonatal resuscitation in first and second level health facilities of Ayacucho and Cusco departments.

### Secondary objectives

To assess intervention and control efficacy in selected health facilities, in order to compare: 1) Time to start positive pressure ventilation after birth; 2) Time to achieve heart rate greater or equal than 100 per minute; 3) Apgar at 1 minute and at 5 minutes; 4) Use of supplemental oxygen after 10 minutes of life; 5) Inspiratory oxygen fraction needed at 30 minutes after birth; 6) Mortality rate during the first 7 days of life; 7) Number of referrals to health facilities with greater resolution capacity during the first 7 days of life; 8) Number of certified health professionals as providers.; and 9) Number of certified health professionals as instructors.

The efficacy of the evaluation will be evaluated at 6 months after the intervention.

Status of the study: This study is ongoing.

Study protocol version 1.0 – March 31th, 2017.

Trial registration data can be found in Supplementary File 1.

## Research methodology and experimental plan

### Settings

Ayacucho and Cusco are two Peruvian regions located to the south of the country. Both regions were selected as thay have a neonatal mortality rate above 15 per 1,000 live births and they are accessible through air travel. These regions cover areas of Andean highlands, but their territory is mainly on the Amazon rainforest (Peru Export and Tourism Promotion Board, 2017; http://www.peru.travel). Also, in those departments there is the “Valley of the Apurímac, Ene and Mantaro Rivers”, also known as the VRAEM, which is extremely poor and one of the major areas of coca growing in Peru ^19^.

According to the Peruvian Registry of Institutions that Provide Health Services (RENIPRESS), Ayacucho has 418 and Cusco 848 registered health facilities ( *Registro Nacional de Instituciones Prestadoras de Servicios de Salud 2017* website; http://www.minsa.gob.pe/portalweb/02estadistica/estadistica_2.asp?sub5=2). However, last year (2016) almost 15,000 deliveries were attended at 80 public health facilities of primary and secondary level, which represents 50% of all deliveries in both regions ( *Sistema de Registro del Certificado de Nacido Vivo en Linea* 2017 website; http://www.minsa.gob.pe/cnv/).

Basic teams are composed by doctors, nurses, midwives and nurse technicians, who are registered in a database updated by the health personnel observatory reports ( *Observatorio de recursos humanos en salud* 2017 website; http://observatorio.inforhus.gob.pe/). This observatory states that, up until June 11 ^th^, 2017, Ayacucho had 337 doctors, 726 nurses, 466 midwives and 743 nurse technicians; while Cusco had 661 doctors, 942 nurses, 486 midwives and 806 nurse technicians.

### Study design

The proposed study design is a randomized cluster trial. The units of study and analysis will be twelve health facilities from Ayacucho and Cusco departments. Each health facility will be a cluster. Clusters will be randomly assigned either to the MP-ICT course and certification package (intervention) or to the standard training and certification package (control). Additional details are shown in Table 1.

**Table 1.** Schedule of enrolment, interventions, assessments and final report: RCPNEOPERU Project.

	STUDY PERIOD									
	Project Management		Allocation	Post-allocation		Pause	Assessments			Close-out
BIMESTER	*B _-2_*	*B _-1_*	*0*	*B _3_*	*B _4_*	*B _5_*	*B _6_*	*B _7_*	*B _8_*	*B _9_*
	*2017-04* *2017-05*	*2017-06* *2017-07*		*2017-08* *2017-09*	*2017-10* *2017-11*	*2017-12* *2018-01*	*2018-02* *2018-03*	*2018-04* *2018-05*	*2018-06* *2018-07*	*2018-08* *2018-09*
**ENROLMENT**										
Ethical approval and Logistics	**X**	**X**								
Allocation			**X**							
Eligibility screen				**X**						
Informed consent				**X**						
**INTERVENTIONS**										
Intervention ICT				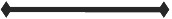						
Standard control				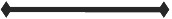						
**ASSESSMENTS**										
Baseline				**X**						
Primary Outcome							**X**	**X**	**X**	**X**
Other Outcomes							**X**	**X**	**X**	**X**
**FINAL REPORT**										**X**

**Legend**: Intervention ICT=Training and certification using Information and Communications Technology **Template reference**: Chan A-W, Tetzlaff JM, Gøtzsche PC, Altman DG, Mann H, Berlin JA, *et al.* SPIRIT 2013 explanation and elaboration: guidance for protocols of clinical trials. BMJ: British Medical Journal. 2013;346.

### Justification for design

The primary target of the intervention is the health provider. However, we made the decision to assess the heart rate of infants as an objective and practical way to evaluate the effect of the intervention on the adequacy of the resuscitation provided by the health worker, in line with other studies targeted primarily to the health provider ^20^. Training will be provided to groups of health professionals who work at a health facility. Also, because of the geographical distribution of health facilities, their selection as units of study helps to minimize the possibility of contamination between interventions.

### Inclusion and exclusion criteria

Primary and secondary level facilities located in Ayacucho and Cusco that have a neonatal mortality rate higher than 15 per 1,000 livebirths will be eligible. Health facilities whose authorities refuse participation of their health professionals, facilities with less than 290 births a year, facilities located at more than 210 kilometers from the department capital, and those located in high-risk areas due to social unrest will be excluded. Health professionals who attend deliveries and accept their voluntary participation will be included after signing informed consent forms.

### Sample size

We must emphasize that our study will be conducted at a pilot scale and thus we considered the minimum sample size for such study level. Assuming the occurrence of about 4,000 births during a period of 6 months of filed study observation, we aim at discriminating a 4% difference in the proportion of newborns with heart rate equal or greater than 100 beats per minute at two minutes of life between the intervention and the control group ^18^. We would require 12 clusters (health facilities) divided in two arms, with six facilities allocated to each arm, and with an average of 334 deliveries per facility during the observation period. The proposed power of the study is 80% and the acceptable alpha error is 5%. The percentage for heart rate >100 bpm at two minutes of life during suitable resuscitation using self-inflated bag is 90% ^21^, and it is the expected value for the intervention group. For the control group, at least 4 percentage points below the value achieved in the intervention group is expected (i.e. no more than 86%).

## Description of interventions

Since the whole health facility will receive the intervention or the standard training, it is unrealistic to expect that we could mask the assessors of the practical component. Thus health facilities or instructors will not be blinded to the type of intervention they receive or provide. The health professionals of each health facility will be informed about the training they will receive after the allocation for Standard or MP-ICT training. We will formally assess the theoretical and clinical skills of health care providers through the theoretical and practical exams that all participants in each group will take.

### Standard training

Standard training will be conducted through a theoretical-practical course, which will be administered once during the study period, to all health professionals of the selected facilities, according to the randomization. It is an 8-hour course, with 3 hours of theory (based on suggested readings) and 5 hours of practice, which will take place during a single day, from 9:00 a.m. to 6:00 p.m. The course will be performed by staff of the Neonatal Unit of the National Institute of Child Health, and will be coordinated by an NRP instructor accredited by the American Academy of Pediatrics. Suggested readings will include the following sequential topics: 1) Initial Steps, 2) Positive Pressure Ventilation, 3) Cardiac Massage, 4) Intubation and Medications, and 5) General Review. Practical sessions will be carried out in groups, with 10 or more people per group, and will include all the health professionals of the facility. The practical content part will consist of simulations based on predetermined scenarios using neonatal mannequins and supplies needed for basic and advanced resuscitation, covering each component of the theoretical content. One instructor will be assigned to each group. A baseline and a follow-up theoretical test will be administered.

Participants completing their attendance to theoretical sessions, participating in the simulated practices and approving the printed exams taken the same day will we granted a Standard Certification. The practice will be evaluated qualitatively in terms of assistance, participation and performance of activities.

### Multi-platform ICT training

The continuous process of training and certification in neonatal resuscitation will be developed as a multi-platform format, rechargeable online and accessible offline, and will be complemented with simulated practices. The preliminary format of this system is already in place, and is based on an interactive web platform ( www.rcpneoperu.org), which was initially developed as part of the activities proposed in another project (Project first breath; http://www.grandchallenges.ca/grantee-stars/0690-01-10, adapted and used with permission). The platforms will contain training packages, and links to review and download neonatal resuscitation tools, technical documents, and regulatory information. During the first three months of the project, the platform will be improved and adjusted to the needs of the fieldwork in Ayacucho and Cusco. The adaptations include increasing hosting, ameliorating usability, uploading packages and videos, improvement of examination and certifications, and tests for readiness. The MP-ICT resource is user-friendly and can be accessed from remote locations through computers, personal portable devices and cell phones. It allows downloading of the different documents, which facilitates learning and theoretical evaluation in an interactive and virtual way, without the presence of instructors or teachers.

Authorized participants will be able to download the information for the theoretical activities in four packages: Package A, B, C and D: each one containing interactive tools as videos and action mazes (as Quandary), and other videos, documents or short text to read in Spanish. Through the website virtual meetings, as forums or chats, can be initiated. The interactivity is expressed in 360° videos, which allows the user to change the observation point in a recorded environment. The interactivity for action mazes, like Quandary (a software application suite for creating action mazes Version 2.3/Windows; http://www.halfbakedsoftware.com/quandary.php) has demonstrated that feedback-oriented interactive exercises increase higher-order cognitive skills ^22^. The learning is completed according to the student’s pace and needs, and the platforms offer didactic material. The learning process can be done in groups, but the exam is online and individual, and requires Internet connection. Staff can be trained to become a provider and then qualify to become an instructor. At least one instructor will be certified at each facility in order to train the trainers for continuing education in every facility. Instructors will be given access to the web platform to include news of their activities and to evaluate the practices of aspiring providers in their health facilities. 

The theoretical online exam consists of 40 questions distributed in four modules encompassing basic and advanced neonatal resuscitation. Basic modules include a) Initial Steps of Resuscitation, and b) Positive Pressure Ventilation. Advanced modules include c) External cardiac massage, and d) Intubation and Medications.

Examination for each module allows unlimited attempts, and it is necessary to achieve at least 60% success to be approved. According to approved modules and practices, health personnel can obtain a supplier or instructor certification in both basic neonatal resuscitation and advanced neonatal resuscitation. Two types of examination have been prepared, according to the objective of the applicant. One is an aimed at applicants to Neonatal Resuscitation Provider. The other one, an advanced exam, is for applicants to Neonatal Resuscitation Instructor.

All participants need to complete both the Theoretical and the Practical training components. The platform also will allow the use of an agenda-type tool for scheduling practices for aspiring providers or instructors who have passed their online exam. The Theoretical Component is available after the candidate has been registered in the multi-platform portal, to which they can access through a PC, tablet or cell phone with Internet connection. Access to the content is online the first time, but it may also be offline, after downloading the corresponding training modules.

The Practical Component can be taken only after the Theoretical Component is approved at the participants’ facilities. It consists of face-to-face simulations of predetermined clinical scenarios using mannequins and the needed equipment (bag and mask, suction bulbs, towels, etc). This component has 5 hours of practice, which will take place from 8:00 a.m. to 1:00 p.m. in the morning, or from 2:00 p.m. to 7:00 p.m. in the afternoon. Staff of the Neonatal Unit of the National Institute of Child Health and/or a local trained instructor will guide the practice. An NRP instructor accredited by the American Academy of Pediatrics will coordinate all practices.

Trained instructors will rate the trainees’ performance on the Practical Component, according to assistance and participation in practical activities and qualitative qualification of approving performance. The Practical Component is intended only for health professionals who approved the theoretical exam online and who scheduled their participation with a selected instructor. Practical simulations will be carried out in groups of 3 to 4 people, and an instructor will be assigned for each group. They do not require theoretical presentations, pre-test or post-tests.

To be granted an MP-ICT Certification the trainee needs to have passed the online theoretical exam and the practical skills assessment.

An example of MP-ICT training can be found in Supplementary File 2.

### Outcomes

Our primary outcome is the percentage of infants with heart rate equal or greater than 100 per minute at two minutes of life. This outcome is an adequate proxy for effective response to neonatal resuscitation measures in the delivery room ^20, 21, 23^.

The secondary outcomes are described in our secondary objectives.

### Randomization

Computer-generated random numbers will be used to assign the selected health facilities to either MP-ICT training (Intervention Group) or to standard training (Control Group). There will be a matching process of health facilities by proportion of groups of non-medical professionals (nurses and obstetricians) and by availability of basic equipment and maternal and newborn care supplies (the information for which will be available to the health regional directorates), to ensure that health facilities are comparable. Once paired, they will be randomly allocated through a blocked randomization, to ensure a balanced distribution of facilities in each group.

### Effectiveness assessment

After randomization of each facility, standard training or MP-ICT training will be conducted. Evaluations will include a baseline assessment of available equipment and supplies for neonatal resuscitation, immediately after enrolment and before training. To determine the duration of the effects of the intervention, the proposed outcome indicators will be evaluated six months after the training. The primary and secondary outcomes will be assessed intrapartum (within 5 minutes after birth) and postpartum (at 24 hours and 7 days, to know the outcome of the infant). The assessments will be based on an observation sheet (see Supplementary File 3), in which trained research assistants (registered nurses or equivalent local personnel) will record the activities conducted during the delivery and the birth, and the results of the neonatal resuscitation. The observation of performance in neonatal resuscitation will also be recorded randomly through videos, to verify the validity of the data collected by the research assistant. The videos will be reviewed by the field monitor and by one of the investigators. In order to comply with confidentiality requirements, the videos will be deleted after verification of information.

### Statistical analysis

An intention to treat analysis will be performed, including all clusters as they were initially assigned, including premature withdrawals. Additionally, a per-protocol analysis will be carried out, where only clusters that have completed the study protocol will be considered. The primary outcome will be compared in each arm using Chi-square test if the variable of distribution is normal, or alternatively through the Fisher’s exact test. Secondary outcomes will be compared using the Student’s t-test or the Kruskal-Wallis test. For supplementary oxygen use, the Chi-square test or Fisher’s exact test will be used.

### Staff training

We will resort to local staff as research assistants, including the field supervisor. A small monetary incentive will be available for field workers and study monitors. They will conduct the preparatory work and the study procedures, including information presentations, informed consent administration, observation of case management, and completion of case report forms. Such personnel will be trained in the conduction of studies complying with the standards of good practice, before the study initiation.

## Ethical aspects and confidentiality

The proposal, instruments and consent forms have been approved by the Institutional Ethics Committee of the Universidad Peruana Cayetano Heredia (record, 273-10-17). The Project Lead will communicate major protocol amendments to relevant parties. Written authorization will be obtained from the authorities of the regional health departments (DIRESAS) involved and from the authorities of the health facilities. Written informed consent will be obtained from the health professionals who will participate in the neonatal resuscitation training ( Supplementary File 4). Written informed consent of the mothers will also be obtained for the collection of birth and newborn data ( Supplementary File 5). On a random basis, the observation of performance in neonatal resuscitation will be recorded in video, which will be deleted after verification of the information. This information is included on the consent forms.

The information obtained will be coded and personal identification data, like names and dates of birth, will be deleted to protect confidentiality. Data access will be limited to the Principal Investigator and authorized Co-Investigators. The study documents will be stored in locked environments at the Centro de Investigación para el Desarrollo Integral y Sostenible (CIDIS) at Universidad Peruana Cayetano Heredia, to which access will be granted only to the Principal Investigator and the Co-Investigators authorized by the Principal Investigator.

## Expected results

The project proposes to streamline the process of training providers and trainers in neonatal resuscitation in Peru, a country with 31 million inhabitants and a current national neonatal mortality rate of 8 per 1,000 livebirths ^9^. However, neonatal mortality amounts 20 per 1,0000 livebirths in rural areas ^6^. This context poses a huge challenge needing innovative interventions that build on previous success factors that allowed Peru to remarkably reduce maternal and neonatal deaths ^5, 6^. We expect that our proposed intervention will show effectiveness amenable to scaling it up at a national level, as part of a national neonatal resuscitation program, while prioritizing the most remote rural areas of the country, who are lagging behind and need urgent action.

We also anticipate that the continuous process of certification of health personnel in remote areas using multi-platform technologies will become a key component of the know-how to develop similar strategies in other low and middle-income countries, where national neonatal resuscitation programs are absent or are still incipient. The MP-ICT strategy is well suited to close the urban-rural gap in maternal and neonatal gap present in these countries, provided its design and implementation are developed, by taking into account an indispensable equity lens.

If the intervention proves to be effective, we will have made substantial progress in interaction with Peruvian policy-makers to ensure a smooth process of incorporation of the MP-ICT strategy into a National Neonatal Resuscitation Training and Certification Program that could be implemented for reaching health personnel at national level. This program would take advantage on the MP-ICT contributions, including the use of its interactive portal ( www.rcpneoperu.org). In the meantime, we aim to reach health care personnel currently registered in Peru, which until June 2017 included 258 neonatologists and 3554 pediatricians, 23,101 general physicians, 28,363 nurses and 13,634 midwives, as well as students of different professional careers (updated Peruvian Physicians Registry http://cmp.org.pe/servicios/conoce-a-tu-medico; and *Observatorio de recursos humanos en salud* 2017 ** http://observatorio.inforhus.gob.pe/).

## Ethical approval

The Institutional Ethics Committee, Universidad Peruana Cayetano Heredia (record 273-10-17; May 4 ^th^, 2017).

## Supplementary material

Supplementary File 1: Trial registration data.

Supplementary File 2: An example of MP-ICT

Supplementary File 3: Observation sheet.

Supplementary File 4: Consent form for health professionals (version 2.0).

Supplementary File 5: Consent form for mothers (version 2.0).

Supplementary File 6: Completed SPIRIT checklist.
