# Supplementary material for: Continuous training and certification in neonatal resuscitation in remote areas using a multi-platform information and communication technology intervention, compared to standard training: A randomized cluster trial study protocol
Source: F1000Res. 2018 Mar 8;6:1599. Originally published 2017 Aug 30. [Version 3] doi: 10.12688/f1000research.12269.3 (PMC6034561; doi:10.12688/f1000research.12269.3)
Supplement: Supplementary file 1 [file f1000research-6-15466-s0000.tgz › 113374ab-5b2d-4224-923c-c33c4ced6528.docx]

**Trial registration data**

- Primary registry and trial identifying number: NCT03210194
- URL: <https://clinicaltrials.gov/ct2/show/NCT03210194>
- Date of registration in primary registry: June 30^th^, 2016
- Secondary identifying numbers: SIDISI - 100651
- Source of monetary or material support: Grand Challenges Canada
- Primary sponsor: Universidad Peruana Cayetano Heredia
- Contact for public queries: Dr. Luis Huicho and Dr. Carlos Delgado
- Contact for scientific queries: Dr. Carlos Delgado and Dr. Luis Huicho
- Public title: RCPNEOPERU project
- Brief title: Randomized Cluster Trial on Innovative and Standard Strategies for Neonatal Resuscitation Training (RCPNEOPERU Project)
- Scientific title: Continuous training and certification in neonatal resuscitation in remote areas using a multi-platform information and communication technology intervention compared to standard training: A randomized cluster trial study protocol
- Country of recruitment: Perú
- Problem studied: Health Care Personnel training alternatives in neonatal resuscitation on remote areas.
- Intervention: Training and certification using a multi-platform information and communication technology intervention
- Key inclusion and exclusion criteria:
  - - Inclusion criteria:
      - Primary and secondary level facilities located in Ayacucho and Cusco that have a neonatal mortality rate higher than 15 per 1,000 live births will be eligible
    - Exclusion criteria:
      - Health facilities whose authorities refuse participation of their health professionals; facilities with less than 290 births a year; facilities located at more than 210 kilometres from the department capital; and those located in high risk areas due to social unrest will be excluded

**Trial registration data (continuation)**

- Study type: cluster randomized trial
- Date of first enrolment: August, 2017
- Target sample size: 12 health centres
- Recruitment status: Not yet recruiting
- Primary outcome: Percentage of infants with heart rate equal or greater than 100 per minute at 2 minutes of life
- Key secondary outcomes: 1) time to start positive pressure ventilation; 2) time to achieve heart rate greater than 100 per minute; 3) Apgar at 1 minute and at 5 minutes; 4) use of supplemental oxygen after 10 minutes of life; 5) inspiratory oxygen fraction needed by the new-born at 30 minutes after birth; 6) early neonatal mortality, will be gathered until to the day 7 of life; 7) number of referrals to health facilities with greater resolution capacity, monitored until the day 7 of life; and 8) number of certified health professionals.
