# Supplementary material for: Continuous training and certification in neonatal resuscitation in remote areas using a multi-platform information and communication technology intervention, compared to standard training: A randomized cluster trial study protocol
Source: F1000Res. 2018 Mar 8;6:1599. Originally published 2017 Aug 30. [Version 3] doi: 10.12688/f1000research.12269.3 (PMC6034561; doi:10.12688/f1000research.12269.3)
Supplement: Supplementary file 2 [file f1000research-6-15466-s0001.tgz › e0724d87-cd11-4568-938c-149c684bd08f.htm]

Reanimación Neonatal


<=
 Index 
=>

## Reanimación Neonatal

Bookmark
Restart

### 

|  |  |
| --- | --- |
| Estimado participante,  Este es una simulación interactiva realizada con el programa Quandary con el propósito de reforzar conocimientos en Reanimación Neonatal.  Esta simulación ha sido elaborada con fines didácticos, tomando en cuenta las guias y recomendaciones más recientes (Oct-2015). Sin embargo, estas simulaciones no han sido elaboradas para orientar decisiones clinicas ni cuidados de pacientes específicos.  Si desea mayor información, por favor comunicarse con:   Carlos A. Delgado MD, PhD  Start |  |

OK

<=
 Index 
=>
