# Supplementary material for: Continuous training and certification in neonatal resuscitation in remote areas using a multi-platform information and communication technology intervention, compared to standard training: A randomized cluster trial study protocol
Source: F1000Res. 2018 Mar 8;6:1599. Originally published 2017 Aug 30. [Version 3] doi: 10.12688/f1000research.12269.3 (PMC6034561; doi:10.12688/f1000research.12269.3)
Supplement: Supplementary file 3 [file f1000research-6-15466-s0002.tgz › 8d17e0dc-bacb-450b-ab4a-792c7b0c3fbb.zip!/Supplementary File 3/Observation sheet Spanish.pdf]

PROYECTO RCPNEOPERU

Código Ej: 0001
Fecha ____________ **Peso del RN** ____________
Nombres y Apellidos de la mamá ____________ **Edad Gestacional** < 37 sem 37-41 sem > 42 sem
N° de Historia Clínica ____________ **Sexo** (Femenino) (Masculino)
Factores de Riesgo (NO) (SI, ESPECIFIQUE) ____________ ____________ ____________

Reloj	Momentos	Cronómetro	Oxímetro de Pulso SatO2	Oxímetro de Pulso Frec-Card	Respiración (Ausente) (Débil) / (Fuerte)	Frecuencia Cardíaca	¿VPP, Masaje, Intubación, Medicamentos? (Anotar hora en cronometro)
00:00	Hora de nacimiento	**00:00**					
		00:10		Anotar Frecuencia Cardiaca ≥ 100 (y hora en Cronómetro)		Anotar Frecuencia Cardiaca ≥ 100 (y hora en Cronómetro)	
		00:20					
		00:30					
		00:40					
		00:50					
	**Un minuto**	**01:00**	**Anotar**	**Anotar**	**Anotar (Ausente) (Débil) / (Fuerte)**	**Contar en 6 seg**	
		01:10		Anotar Frecuencia Cardiaca ≥ 100 (y hora en Cronómetro)		Anotar Frecuencia Cardiaca ≥ 100 (y hora en Cronómetro)	
		01:20					
		01:30					
		01:40					
		01:50					
	**Dos minutos**	**02:00**	**Anotar**	**Anotar**		**Contar en 6 seg**	
		02:10		Anotar Frecuencia Cardiaca ≥ 100 (y hora en Cronómetro)		Anotar Frecuencia Cardiaca ≥ 100 (y hora en Cronómetro)	
		02:20					
		02:30					
		02:40					
		02:50					
	**Tres minutos**	**03:00**	Anotar	Anotar			
		03:10		Anotar Frecuencia Cardiaca ≥ 100 (y hora en Cronómetro)		Anotar Frecuencia ≥ 100 (y hora en Cronómetro)	
		03:20					
		03:30					
		03:40					
		03:50					
	**Cuatro minutos**	**04:00**	Anotar	Anotar			
		04:10		Anotar Frecuencia ≥ 100 (y hora en Cronómetro)		Anotar Frecuencia Cardiaca ≥ 100 (y hora en Cronómetro)	
		04:20					
		04:30					
		04:40					
		04:50					
	**Cinco minutos**	**05:00**	**Anotar**	**Anotar**	**(Ausente) (Débil) / (Fuerte)**	**Contar en 6 seg**	

Gestos	Tono	Piel
Nada<br>Débil<br>Fuerte	Nada<br>Flexión<br>Movimiento	Cianosis<br>Acrocianosis<br>Normal

Gestos	Tono	Piel
Nada<br>Débil<br>Fuerte	Nada<br>Flexión<br>Movimiento	Cianosis<br>Acrocianosis<br>Normal

Marcar la Ubicación del Bebé a las 24 horas:

(Alta) (Alojamiento Conjunto) (Hospitalización) (Transferencia) (Fallecido)

Marcar la Ubicación del Bebé a los 7 días de vida:

(Alta) (Alojamiento Conjunto) (Hospitalización) (Transferencia) (Fallecido)
