# Supplementary material for: Continuous training and certification in neonatal resuscitation in remote areas using a multi-platform information and communication technology intervention, compared to standard training: A randomized cluster trial study protocol
Source: F1000Res. 2018 Mar 8;6:1599. Originally published 2017 Aug 30. [Version 3] doi: 10.12688/f1000research.12269.3 (PMC6034561; doi:10.12688/f1000research.12269.3)
Supplement: Supplementary file 4 [file f1000research-6-15466-s0003.tgz › e6333ffe-88b7-4cba-bf54-4d9a34ce7dba.zip!/Supplementary File 4/Consent form for health professionals English translation.docx]

**INFORMED CONSENT - STAFF**

Authorization of Health Personnel to participate in training and evaluation of their performance in neonatal resuscitation

**Consent to participate in a research study on the care of health personnel trained in neonatal resuscitation**

______________________________________________________________________

Institutions	: Universidad Peruana Cayetano Heredia

Researchers	: Luis Huicho, Carlos Delgado

Title	: Continuous training and certification in neonatal resuscitation in remote areas using a multi-platform information and communication technology intervention, compared to standard training: A randomized cluster trial

______________________________________________________________________

**Purpose of Study**

We are inviting you to participate in a study called: "Continuous training and certification in neonatal resuscitation in remote areas using a multi-platform information and communication technology intervention, compared to standard training: A randomized cluster trial" This is a study developed by researchers from the Universidad Peruana Cayetano Heredia with the support of the Canadian Government.

Currently, newborns can present asphyxia at birth, due to uncontrolled pregnancies, prenatal risk factors or inadequate training of health personnel. Lack of time and work constraints may hamper opportunities of health personnel for receiving continuous training.

 A big problem is that it is difficult to train health workers who work in distant areas. In this project we aim to train clinical staff of health facilities in Ayacucho and Cusco, so they can manage in a better way the strategies of Neonatal Resuscitation. One group of health workers of health facilities will receive training multiplatform information and communication technologies and the other group will be receive conventional training. We aim in this way to evaluate the best way to train health personnel in neonatal resuscitation. We expect that this knowledge will enable us to implement a National Neonatal Resuscitation Program in remote areas of our country.

We need to study how the training of health personnel can be improved using modern computer technologies.

We want to evaluate how the training programs in different locations in Ayacucho and Cusco can teach us the best way to implement these strategies in other parts of the country, improving the quality of care of newborns, even in the most remote areas of the country.

**Procedures**

If you agree to participate in this study, the following activities will be carried out:

1. This year, you may participate in the Neonatal Resuscitation Training Program to be held at your health facility. You may receive training using multiplatform technologies or you may be trained using conventional training. We will randomly assign the health facilities to the either the Multiplatform or the Conventional training. Mixed training will not be performed. Participation of health workers will include carrying out the theoretical and practical academic components to successfully complete the training in neonatal resuscitation.
2. Six months after training, your performance will be assessed through a structured observation of how you deliver childbirth care. we ask you to allow us observing and recording minute-by-minute, everything that happens during the first 5 minutes within birth, as well as identifying what happened to the baby 24 hours and 7 days after birth. In some cases and in a random way, observation of performance in neonatal resuscitation will be recorded through video, to evaluate the validity of the data collected by the study team. The videos will be checked by the field monitor and by one of the investigators, and will be deleted after verification of the information.

**Risks**

There is no risk involved in your participating in this research. However, some questions can cause discomfort. You are free to answer them or not.

**Benefits**

There is no direct benefit to you from participating in this study. However, you will be informed in a personal and confidential manner of the results obtained from the Observation Sheet. The Form to be completed is an initial assessment of delivery care and is not a diagnosis. If this is the case, you will be advised to refer to a specialist. Your participation in this study will have no cost to you. In the event that a newborn requires additional assistance, it will be suggested to follow the recommendations of the professionals in charge for referral of the newborn to pediatric or neonatal services of the Regional Hospital at the Capital of the Department.

**Costs and incentives**

You will not have to pay anything to participate in the study. You will not receive any economic or other incentive, only the satisfaction of collaborating to a better understanding of the way we perform the training of our health personnel in neonatal resuscitation strategies.

**Confidentiality**

We will store your information with codes and not with names. If the results of this follow-up are published, no information will be displayed to identify the persons participating in this study. Your records will not be shown to anyone outside the study without your consent.

**Rights of the participant**

If you choose to participate in the study, you can withdraw from it at any time. You may also choose not to participate in a part of the study, without any resulting consequence. If you have any further questions, please ask the study staff, or call Researcher Carlos Delgado to phone number 940222137.

If you have questions about the ethical aspects of the study, or think you have been treated unfairly, you may contact Dr. Frine Salmavides, Chairwoman of the Institutional Research Ethics Committee of the Universidad Peruana Cayetano Heredia, to phone 01- 319000, annex 2271.

**CONSENT**

I voluntarily agree to participate in this study. I understand what will happen to me if I participate in the project, I also understand that I can choose not to participate, and that I can withdraw from the study at any time.

My consent includes authorization to make recordings: YES (	)	NO (	)

_____________________________________	_______________________

Participant	Date

First name:

ID:

_____________________________________	_______________________

Witness	Date

First name:

ID:

_____________________________________	_______________________

Investigator	Date

First name:

ID:
