# Supplementary material for: Continuous training and certification in neonatal resuscitation in remote areas using a multi-platform information and communication technology intervention, compared to standard training: A randomized cluster trial study protocol
Source: F1000Res. 2018 Mar 8;6:1599. Originally published 2017 Aug 30. [Version 3] doi: 10.12688/f1000research.12269.3 (PMC6034561; doi:10.12688/f1000research.12269.3)
Supplement: Supplementary file 4 [file f1000research-6-15466-s0003.tgz › e6333ffe-88b7-4cba-bf54-4d9a34ce7dba.zip!/Supplementary File 4/Consent form for health professionals Spanish.pdf]

CONSENTIMIENTO INFORMADO - PERSONAL

Autorización del Personal de Salud para participar en el entrenamiento y evaluación de su desempeño en reanimación neonatal

Consentimiento para participar en un estudio de investigación sobre la atención del personal de salud entrenado en reanimación neonatal

---

Instituciones	:	Universidad Peruana Cayetano Heredia
Investigadores	:	Luis Huicho, Carlos Delgado
Título:		Capacitación y certificación continúa en reanimación neonatal en zonas alejadas a través de tecnología multiplataforma: Un ensayo aleatorio por conglomerados

---

Propósito del Estudio:

Invitamos a Usted a participar en un estudio llamado: “Capacitación y certificación continúa en reanimación neonatal en zonas alejadas a través de tecnología multiplataforma: Un ensayo aleatorio por conglomerados”. Este es un estudio desarrollado por investigadores de la Universidad Peruana Cayetano Heredia con apoyo del Gobierno Canadiense.

En la actualidad, la atención de cualquier recién nacido puede llevar a casos de Asfixia al Nacer, debido a embarazos sin control, factores de riesgo prenatal o entrenamiento del personal de salud, que predisponen a su aparición. El tiempo y las condiciones de trabajo no siempre facilitan mantener al personal de salud en continua capacitación.

Un gran problema es que es difícil realizar el entrenamiento del personal de salud que trabaja en zonas un poco alejadas. En este proyecto hemos entrenado al personal de Centros de Salud y Hospitales de Apoyo en Ayacucho y Cusco, para que tengan un mayor dominio de estrategias de Reanimación Neonatal. Para la capacitación, en unos establecimientos de salud vamos a usar tecnologías multiplataforma con y sin acceso a internet y en otros establecimientos de salud se realizará entrenamiento convencional. El propósito de esta investigación es evaluar cuál es la mejor manera de entrenar al personal de salud en reanimación neonatal, y esperamos que ese conocimiento nos permita implementar un Programa Nacional de Reanimación Neonatal en zonas alejadas de nuestro país.

Es por lo mostrado que creemos que es necesario estudiar cómo se puede mejorar el entrenamiento del personal de salud usando tecnologías modernas de informática.

Queremos evaluar cómo los programas de capacitación en diferentes localidades de Ayacucho y Cusco pueden enseñarnos la mejor manera de implementar estas estrategias en otros lugares del país, mejorando la calidad de atención de los recién nacidos, incluso en los ámbitos más alejados de nuestro Perú.

COMITE INSTITUCIONAL DE ETICA
UNIVERSIDAD PERUANA CAYETANO HEREDIA DUICT
CIE
APROBADO
F. APROBACIÓN: 04/05/17

Procedimientos:

Si usted acepta participar en este estudio se llevarán a cabo las siguientes actividades, con su participación:

1. En el curso de este año, Usted podrá participar en el Programa de Entrenamiento en Reanimación Neonatal que se realizará en su Establecimiento de Salud. Puede recibir entrenamiento usando tecnologías multiplataforma o será entrenado usando el entrenamiento convencional. La selección de lo que corresponderá a cada establecimiento (Tecnologías multiplataforma o Entrenamiento Convencional), se realizará previamente, en forma aleatoria. No se realizarán entrenamientos mixtos. Su participación consiste en dedicarse a completar los objetivos académicos teóricos y prácticos para completar con éxito el entrenamiento en reanimación neonatal.
2. Seis meses después del entrenamiento, se evaluará su desempeño a través de una observación estructurada de la forma en que realiza la atención de partos. Su participación consiste en permitirnos observar y registrar minuto a minuto, todo lo que ocurre durante los primeros 5 minutos desde el nacimiento, así como identificar dónde se encuentra el bebé 24 horas y 7 días después. En algunos casos, la observación del desempeño en la reanimación neonatal será registrada en video en forma aleatoria, con el fin de establecer medios de verificación que permitan evaluar la validez de los datos recogidos por el trabajador de campo. Los videos serán revisados por el monitor de campo y por uno de los investigadores y serán borrados luego de la verificación de información.

Riesgos:

No existe ningún riesgo al participar de este trabajo de investigación. Sin embargo algunas preguntas le pueden causar incomodidad. Usted es libre de responderlas o no.

Beneficios:

No existe beneficio directo para Usted por participar de este estudio. Sin embargo, se le informará de manera personal y confidencial de los resultados que se obtengan de la Ficha de Observación realizada. La Ficha que se completará, es una evaluación inicial de la atención del parto y no es un diagnóstico. De ser el caso, se le recomendará para que acuda a un especialista. Su participación en este estudio no tendrá ningún costo para usted. En caso de que algún recién nacido requiera asistencia adicional a la recibida en el establecimiento de salud, se sugerirá seguir las recomendaciones de los profesionales a cargo para una referencia a servicios de Pediatría o Neonatología del Hospital Regional en la respectiva Capital del Departamento.

Costos e incentivos

Usted no deberá pagar nada por participar en el estudio. Igualmente, no recibirá ningún incentivo económico ni de otra índole, únicamente la satisfacción de colaborar a un mejor entendimiento de la forma en que realizamos el entrenamiento y capacitación de nuestro personal de salud en estrategias de reanimación neonatal.

Confidencialidad:

Nosotros guardaremos su información con códigos y no con nombres. Si los resultados de este seguimiento son publicados, no se mostrará ninguna información que permita la identificación de

COMITE INSTITUCIONAL DE ETICA
UNIVERSIDAD PERUANA CAYETANO HEREDIA
DUICT
CIE
APROBADO
F. APROBACIÓN: 04/05/17

las personas que participan en este estudio. Sus archivos no serán mostrados a ninguna persona ajena al estudio sin su consentimiento.

Derechos del participante:

Si usted decide participar en el estudio, puede retirarse del mismo en cualquier momento, o no participar en una parte del estudio, sin perjuicio alguno. Si tiene alguna duda adicional, por favor pregunte al personal del estudio, o llamar al Investigador **Carlos Delgado** al teléfono **940222137.**

Si usted tiene preguntas sobre los aspectos éticos del estudio, o cree que ha sido tratado injustamente puede contactar a la Dra. Frine Salmavides, Presidenta del Comité Institucional de Ética en Investigación, de la Universidad Peruana Cayetano Heredia, al teléfono 01- 3190000, anexo 2271.

CONSENTIMIENTO

Acepto voluntariamente participar en este estudio. Comprendo qué cosas me van a pasar si participo en el proyecto, también entiendo que puedo decidir no participar y que puedo retirarme del estudio en cualquier momento.

Mi consentimiento incluye autorización para realizar grabaciones de video: SI ( ) NO( )

Participante | **Fecha**
Nombre:

DNI:

Testigo | **Fecha**
Nombre:
DNI:

Investigador | **Fecha**
Nombre:

DNI:

COMITÉ INSTITUCIONAL DE ÉTICA
UNIVERSIDAD PERUANA CAYETANO HEREDIA DUICT
CIE
APROBADO
F. APROBACIÓN: 04/05/17
