# Supplementary material for: Continuous training and certification in neonatal resuscitation in remote areas using a multi-platform information and communication technology intervention, compared to standard training: A randomized cluster trial study protocol
Source: F1000Res. 2018 Mar 8;6:1599. Originally published 2017 Aug 30. [Version 3] doi: 10.12688/f1000research.12269.3 (PMC6034561; doi:10.12688/f1000research.12269.3)
Supplement: Peer review history

# Peer review: Referee response for version 3

**DOI:** 10.5256/f1000research.15466.r31655

**Published:** 2018-04-09

**Authors:** Sajid Soofi, Shabina Ariff

**Affiliations:** 1 Department of Pediatrics and Child Health, Aga Khan University, Karachi, Pakistan; 2 Department of Pediatrics, Aga Khan University, Karachi, Pakistan

We have no further comments.

We have read this submission. We believe that we have an appropriate level of expertise to confirm that it is of an acceptable scientific standard.

# Peer review: Referee response for version 2

**DOI:** 10.5256/f1000research.14195.r30501

**Published:** 2018-02-20

**Authors:** Sajid Soofi, Shabina Ariff

**Affiliations:** 1 Department of Pediatrics and Child Health, Aga Khan University, Karachi, Pakistan; 2 Department of Pediatrics, Aga Khan University, Karachi, Pakistan

Overall, an interesting topic with implications for developing interventions to improve neonatal mortality. Still neonatal mortality is high in developing country specially in south Asia. This training methodology has significant public health implications. I have few comments.

- Investigators should also assess & compare theoretical and clinical skill of health care providers as direct impact of intervention.
- Design effect should be considered for sample size estimation as health facilities may have diverse situation and availability of facilities.
- Investigators should share statistical plans in details and also consider linear regression model to observe association of different factors and confounders.

We have read this submission. We believe that we have an appropriate level of expertise to confirm that it is of an acceptable scientific standard, however we have significant reservations, as outlined above.

# Response

**Published:** 2018-03-03

**Authors:** Carlos Delgado

**Affiliations:** Universidad Nacional Mayor de San Marcos, Peru

**Response to the referees, March 3th, 2018:**

Carlos Delgado, Universidad Nacional Mayor de San Marcos, Peru

Luis Huicho, Universidad Peruana Cayetano Heredia, Peru

We appreciate the reviewer's comments.

In the following paragraphs we respond to specific observations that we highlight in quotes.

**“Investigators should also assess & compare theoretical and clinical skill of health care providers as direct impact of intervention”**

Thanks for this remark. Actually, we are going to measure both the theoretical and clinical skill of health workers as part of each training way (experimental and control), as we emphasize in the Description of Interventions section. We added a paragraph at the end of this section to further emphasize that we will assess and compare the theoretical knowledge and clinical skill of health workers.

**“Design effect should be considered for sample size estimation as health facilities may have diverse situation and availability of facilities.”**

In the Randomization section we make clear that *"There will be a matching process of health facilities by proportion of groups of non-medical professionals (nurses and obstetricians) and by availability of basic equipment and maternal and newborn care supplies (the information for which will be available to the health regional directorates), to ensure that health facilities are comparable. Once paired, they will be randomly allocated through a blocked randomization, to ensure a balanced distribution of facilities in each group."* We think therefore that we have already taken into account the design effect through these procedures.

**“Investigators should share statistical plans in details and also consider linear regression model to observe association of different factors and confounders.”**

We think that the statistical analyses are described in sufficient detail in the Statistical Analysis section. It is expected that a cluster level analyses may provide inferences equivalent to those obtained using a linear regression model.

# Peer review: Referee response for version 2

**DOI:** 10.5256/f1000research.14195.r27780

**Published:** 2017-12-07

**Authors:** Waldemar A. Carlo

**Affiliations:** 1 Department of Pediatrics, The University of Alabama at Birmingham, Birmingham, AL, USA

The manuscript has been improved based on the reviewer’s comments.

I have read this submission. I believe that I have an appropriate level of expertise to confirm that it is of an acceptable scientific standard.

# Peer review: Referee response for version 1

**DOI:** 10.5256/f1000research.13283.r25481

**Published:** 2017-09-28

**Authors:** Waldemar A. Carlo

**Affiliations:** 1 Department of Pediatrics, The University of Alabama at Birmingham, Birmingham, AL, USA

**General comments**

 This is a cluster randomized controlled trial to compare two educational techniques for NRP training. The sample size is rather small even though the outcome measures are surrogates rather than major outcome measures.

**Background**

 The NRP program is already web-based. The knowledge part is completed on line with subsequent local skills training. This is not clear from the facts given in the Background section.

 It is stated that the comparator background section that training has to be done in the capital city but these training programs usually have local hospital based instructors. A local based instructor is used in all Helping Babies Survive programs and would be the best comparator.

 The study design states that the objective of the intervention is the health care provider not the individual patient. However, the sample size is based on patient outcomes so this is unclear.

 Training will be done in groups of 10 or more. The group is larger than the usual recommended by resuscitation programs.

 The masking of the assessors of the practical component is not addressed.

 The sample size does not take into consideration that only about 10% of the babies will need resuscitation. Comparable studies such as the study by Szyld quoted by the authors have had 10 times as many patients or even more.

 Other countries such as Chile introduce NRP as a nationwide program and reported decreases in neonatal mortality  using standard methods for training.

I have read this submission. I believe that I have an appropriate level of expertise to confirm that it is of an acceptable scientific standard, however I have significant reservations, as outlined above.

# Response

**Published:** 2017-11-01

**Authors:** Carlos Delgado

**Affiliations:** Universidad Nacional Mayor de San Marcos, Peru

We appreciate the reviewer's comments.

 This evaluation is done by comparing two forms of training: The innovative way is to encourage self-learning participation through information and communication technologies, where participants can review the theory and then give an online exam. Self-learning includes strategies such as videos, action mazes and a virtual reality 360 video, only available for logged trainees <https://youtu.be/-rhMJWqijbU>. The traditional form has been called "Standard", but doing so does not take into account that there is no official program and there are no local instructors that have been implemented to train health personnel. Maybe it's better to call this training as “Traditional” instead of “Standard”.

 There is no Neonatal Resuscitation Training Program in the area, although from time to time some local efforts are made for training with various origins and formats (even some training are not updated). The control comparison could have been not to give training, but in order to offer a minimum training, a traditional, face-to-face and mainly expository form was proposed.

 In the following paragraphs we respond to specific observations that we highlight in quotes.

***"The NRP program is already web-based. The knowledge part is completed on line with subsequent local skills training. This is not clear from the facts given in the Background section."***

 Thanks you for your observation. We have edited the third paragraph of the **Background** section to clarify this.

***"It is stated that the comparator background section that training has to be done in the capital city but these training programs usually have local hospital based instructors. A local based instructor is used in all Helping Babies Survive programs and would be the best comparator."***

 We agree that these programs can be taught by a local based instructor, which is our goal. However, we are going to use hospital based instructors because most of the deliveries in our study region are being attended by nurses and midwifes and there is a scarcity of local doctors who can be used as local based instructors. We have clarified this in the second paragraph of the **Choice of Comparator** section.

***"The study design states that the objective of the intervention is the health care provider not the individual patient. However, the sample size is based on patient outcomes so this is unclear."***

 Thanks for the observation. While it is true that the primary target of the intervention is the health care provider, we used heart rate of babies as a quick reflection of the adequacy of the resuscitation provided by the health worker, in line with other interventions targeted primarily at providers to improve their resuscitation performance (please, see ref. 23). We have clarified this in the **Justification for design** section.

***"Training will be done in groups of 10 or more. The group is larger than the usual recommended by resuscitation programs."***

 We agree with your observation. It is our goal to have better trainer/trainee ratios. We were just trying to be realistic in terms of the availability of our resources. We are happy to inform that, once we have started the field work, we have been able to keep the trainer/trainee ratios lower than the expected originally, as we have had more trainers available to teach the program.

***"The masking of the assessors of the practical component is not addressed."***

 Since the whole health facility is considered for receiving th intervention or the standard training, it is unrealistic to expect that we could mask the assessors of the practical component. We have added a statement that clarifies this in the **Description of the Interventions** section.

***"The sample size does not take into consideration that only about 10% of the babies will need resuscitation. Comparable studies such as the study by Szyld quoted by the authors have had 10 times as many patients or even more."***

 Actually, our study is being conducted at a pilot scale. Thus we are looking for the minimum sample size for such study level. Accordingly, we have selected 12 paired health facilities, 6 on each arm and made our sample size calculation, as described in the paper. We clarified this paragraph in the revised paper.

***"Other countries such as Chile introduce NRP as a nationwide program and reported decreases in neonatal mortality using standard methods for training."***

 We agree with your comment. Our long-term goal is for the neonatal certification training and certification program using ICT to become a nationwide program. We have stated this in **Expected Results** section.
